# Renewed Perspectives on the Deep Roots and Broad Distribution of Animal Consciousness

**DOI:** 10.3389/fnsys.2020.00057

**Published:** 2020-08-13

**Authors:** Louis N. Irwin

**Affiliations:** University of Texas at El Paso, El Paso, TX, United States

**Keywords:** cognition, awareness, evolution, arthropods, cephalopods, vertebrates

## Abstract

The vast majority of neurobiologists have long abandoned the Cartesian view of non-human animals as unconscious automatons—acknowledging instead the high likelihood that mammals and birds have mental experiences akin to subjective consciousness. Several lines of evidence are now extending those limits to all vertebrates and even some invertebrates, though graded in degrees as argued originally by Darwin, correlated with the complexity of the animal’s brain. A principal argument for this view is that the function of consciousness is to promote the survival of an animal—especially one actively moving about—in the face of dynamic changes and real-time contingencies. Cognitive ecologists point to the unique features of each animal’s environment and the specific behavioral capabilities that different environments invoke, thereby suggesting that consciousness must take on a great variety of forms, many of which differ substantially from human subjective experience.

## Introduction

Ever since Darwin, students of animal behavior and the nervous system have generally regarded consciousness as a product of the brain, subject to the influence of natural selection (Richards, 1987). Just as the complexity of brains varies across the full range of animal taxa, Darwin (1871) and his contemporary, Romanes (1883), argued that cognition has emerged in grades of complexity over the evolutionary history of animals.

Notwithstanding this early biological approach to mental phenomena, the scientific study of consciousness entered into a hiatus during the first half of the 20th century, accentuated by the rise of genetics (Richards, 1987), the turn toward behaviorism in psychology (Griffin, 1984), and some difficult philosophical problems (Nagel, 1974; Chalmers, 1995; Dennett, 2017). In recent years, however, neuroscientists have started applying their increasingly sophisticated techniques to the study of mental activity, and neurobiologists have resumed consideration of what ecology, ethology, and evolutionary history have to suggest about the original contention of Darwin and his contemporaries that consciousness is a function of the brain molded by natural selection (Churchland, 2007; Engel, 2010).

Perhaps because consciousness is so often thought of as a *thing* instead of a *process* (Rose, 1976), it most often is expressed as a binary possibility—either it exists or it doesnot (Chaisson, 1987; Humphrey, 1992; LeDoux, 2019). And among those animals capable of it, consideration is seldom given to varying degrees or alternative modes of consciousness. The goal of this article is to briefly review the perspectives from cognitive ecology and comparative neurobiology that suggest a return to the view of Darwin and his contemporaries is warranted, and to point out that if indeed consciousness has been forged through natural selection to be suited uniquely to the environment the animal inhabits, logic suggests that it must be multimodal, occurring in highly variable forms largely remote from human experience.

## Definition

Of the modern attempts to define consciousness, I prefer that of John (2003) who described it as “the subjective awareness of momentary experience interpreted in the context of personal memory and the present state.” The terms “awareness” and “experience” are themselves difficult to define without circular references, and “subjective” necessarily invokes phenomenological (personal) experience resistant to objectivization, but all of them can plausibly be attributed to at least some animals with nervous systems of sufficient complexity.

Four features of consciousness deemed irreducible by Feinberg (2012) serve as useful elaborations of John’s definition. He posited that consciousness is: (1) *referential*, or experienced as occurring outside the head; (2) *unified*, or perceived as coherent scenes, sensations, events, or emotions; (3) *qualitatively variable*, consisting of sensory gradations and variations within modalities (as in colors, sounds, intensities, etc.); and (4), *causational*, in its ability to trigger subsequent mental activity and affect behavior.

To summarize by integrating John’s definition with Feinberg’s features, *consciousness is the personal awareness of unified and qualitatively textured current or recalled experience, perceived as existing in the animal’s external or bodily environment, with the capacity to induce further mental activity and/or behavioral action*.

## Nature and Function

Any argument about the origin and varieties of consciousness must begin with a consideration of its nature and function. From clinical observations and personal experience, human consciousness appears in gradations from marginal awareness to full and focused attention. It should be noted, however, that attention and consciousness are not the same things. Stimuli can be attended to unconsciously, and subjects can be conscious of experiences that they are not attending to (Koch and Tsuchiya, 2007).

In the literature of comparative animal psychology, references to roughly three degrees of consciousness are common. The first degree arises from the detection of physical stimuli at the body’s periphery or interior, capable of eliciting an adaptive reflex. This is referred to as “primary” (Edelman, 2003), or “sensory” (Feinberg and Mallatt, 2016), consciousness. It requires some level of arousal or vigilance to make the animal receptive to stimulation and capable of motor activity but does not necessarily imply rich, textured, or complex content. Some authors consider awareness of feelings or affect as distinguishable from but comparable in degree to sensory consciousness (Feinberg and Mallatt, 2016). The second degree of consciousness is self-awareness (Churchland, 2002; Lou et al., 2020), which Churchland (2013) argued is just another form of perception. The highest degree of consciousness is meta-cognition or the conscious knowledge that individuals have about their cognitive capacities (Smith et al., 2003; Al Banna et al., 2016). The second and third levels of consciousness do imply increasingly complex content. As used in this article, consciousness refers to its primary form (both sensory and affective), unless stated otherwise.

A common approach to discerning the function of consciousness is to focus on the key features of subjective experience: its stabilizing properties and qualitative richness, dynamic integration, situatedness, and intentionality (Pennartz et al., 2019). James (1884), for instance, held that consciousness serves to direct attention and dampen chaotic cerebral activity. He viewed it as a means of focusing on one of several simultaneously possible objects or trains of thought (James, 1890). As he noted, “My experience is what I agree to attend to” (James, 1884). A modern version of the same view was expressed by Edelman (2003) that “the neural systems underlying consciousness arose to enable high-order discriminations in a multidimensional space of signals, ” and qualitative differences in content (qualia) provide a basis for those discriminations.

Many authors have seen the integrative nature of consciousness as central to its function (Edelman, 2003; Tononi, 2012; Miyahara and Witkowski, 2019). In some cases, the integration is directed toward managing the multiplicity of incoming sensory information. In others, it is seen as essential for accessing memory stores with which it evaluates the context and significance of incoming information. Edelman saw consciousness as the integration of perceptual and motor events together with memory to construct a multimodal scene in the present (Edelman, 2003). For Pennartz et al. (2019), the biological function of consciousness is to present the subject with a multimodal, situational survey of the surrounding world and body, subserving complex decision-making, and goal-directed behavior.

Consciousness is particularly important for evaluating real-time changes in the situation of a mobile animal behaving appropriately in its environment (Merker, 2005). Indeed, the essence of consciousness for some authors revolves around its role in evaluating appropriate actions to take in given circumstances, anticipating the consequences of those actions, and updating the animal’s position and orientation as it moves through space (Churchland, 2002; Engel, 2010; Clark, 2016; Buzsáki, 2019). Griffin (1984) asserted that an animal is conscious if it is aware of what it is doing or intending to do. As an appreciation for the central role of place in the cognitive landscape has grown (Irwin and Irwin, 2020), so has a realization that movement through its milieu is largely how an animal mentally creates the dimensions of its environment (Merleau-Ponty, 1945; Sheets-Johnstone, 1999).

Every hypothesis about the function of consciousness has at its core the view that it enables the animal to make optimal use of the information available to it, which has obvious survival value. This is not to assert that consciousness is necessary for every beneficial activity—adaptive behaviors from the tropisms of unicellular microbes to complex instinctual behaviors in vertebrates—can occur without conscious reflection. But the ability to maximize the utility of information, integrate it with memory, and focus on elements most critical for survival, either in the moment or over a longer-term, is a biological capability susceptible to favorable natural selection.

## Cognitive Ecology

Jakob Von Uexküll (1926) was an early proponent of the argument that organisms experience life in terms of species-specific, spatio-temporal, “self-in-world” frames of reference. An organism creates and reshapes its perception. Consequently, the minds of different organisms differ, which follows from the individuality and uniqueness of the history of every single organism. Gibson (1977) coined the term *affordance* to denote what the environment uniquely makes possible for any given species. Many observers have noted the need for an animal’s perception and cognition to match the affordances of its environment. Gallagher (1997) argued that “Consciousness and the brain develop and function within a form of existence that is already defined by the world it inhabits.” Anderson (2014) observed that neural organization should be understood in terms of “the brain’s differential propensities to influence the organism’s response to the various features or affordances in its environment.”

There is strong evidence that brains have evolved to respond to environmental pressures (Marino, 2005). Various measures of brain size are positively correlated with: (1) feeding innovation, learning, and tool use; (2) size of behavioral repertoire; (3) social complexity; (4) dietary complexity; and (5) unpredictability of the environment. Ecological principles like the unpredictability of resources in space and time may drive different types of cognition (Lefebvre and Sol, 2008).

An alternative to the influence of environmental variation is that the complexity of the environment in general rather than the unique features of different environments determines the nature of consciousness (Shumway, 2008; Sol, 2009; Mettke-Hofmann, 2014). However, brain evolution does not necessarily proceed from simple to complex, small to large, or diffuse to differentiated (Bullock, 1984; Kaas, 2002). Marino (2005) believes that brain evolution relates more to environmental change than to its complexity. On the other hand, neural and behavioral complexity are clearly correlated (Parker and McKinney, 1999; Neubauer, 2012). So the role that complex environments play in shaping the nature of consciousness cannot be dismissed. Nonetheless, a symposium on the evolution of neurobiological specializations in mammals concluded that there are no simple brains; that brains reflect ecology (Marino and Hof, 2005). Ethologists and comparative animal psychologists have echoed this perspective. Hodos (1986), for instance, argued that natural selection optimizes mechanisms of perception maximally appropriate for the ecological demands of each species, rather than the complexity of information processing in a general sense.

Not everyone has embraced an emphasis on ecological determinants of cognition. Macphail and Bolhuis (2001) claimed to find no convincing support from an ecological account of cognition. In fact, their argument seems to focus on mechanisms of learning specifically more than on consciousness (Bolhuis, 2015). The fact is that all animals exist in an ecological setting, and to the extent that natural selection responds to ecological mandates (Marino, 2005; Sol et al., 2005), it is reasonable to assume that the nature of an animal’s conscious awareness must be attuned to its environmental setting.

## Neurobiology

While evolutionary “gradations” in consciousness have been advocated since the time of Darwin (1871) and Romanes (1883), consciousness has usually been discussed as a bimodal phenomenon, with some evolutionary threshold needing to be achieved for consciousness in an-all-or-none fashion to appear (Jaynes, 1976; Humphrey, 1992; LeDoux, 2019). But neuroanatomy, neurochemistry, and adaptive behavior are not discontinuous over evolutionary time scales, so the sudden emergence of consciousness where it did not previously exist is not plausible. Rather, a gradual increase in resolution and complexity of awareness, coupled with increasing integration of perception with memory as brains became larger and more complex during phylogeny, is logically more persuasive (Rose, 1976; Bullock, 1984; Griffin, 1984; Tononi, 2004; Tononi and Koch, 2015; Mercado, 2008; Koch, 2012; Kiverstein and Rietveld, 2018).

This does not mean that discontinuities in the degree of consciousness do not exist across some phyletic lines. Stark differences in the complexity of consciousness between invertebrates and vertebrates, birds and mammals compared to the other vertebrate Classes, and especially between humans and non-human primates, are generally assumed. What neurobiological evidence can be advanced in support of any of these assumptions?

Feinberg and Mallatt (2016) have provided the most detailed and sophisticated analysis of the occurrence of consciousness across the animal kingdom. They posit seven indicators of sensory consciousness (a large number of neurons, three or more synaptic relays before the pre-motor center, isomorphic organization, reciprocal interactions, multisensory convergence, a neuroanatomical locus for memory, and a selective attention mechanism), as well as five indicators of affective consciousness (operantly learned response to reward or punishment, the behavioral distinction between good and bad outcomes, frustration behavior, self-delivery of analgesics, and approach to reinforcing drugs). Using these criteria, Feinberg and Mallatt (2016) propose the likelihood that some arthropods (especially among the insects and crustaceans), the cephalopods (octopi, squids, nautiloids), and all vertebrates are conscious. While others—especially those focused on the assessment of meta-cognition in mammals and birds—are more skeptical (Humphrey, 1992; Butler, 2008; LeDoux, 2019), the capacity of all vertebrates to experience primary and affective consciousness has widespread support (Griffin, 1984; Koch, 2012; Feinberg and Mallatt, 2016).

Because the neurobiology of all the vertebrates is known in much greater detail than for the invertebrates, distinctions among the vertebrates can be discerned. For example, brain mass relative to body mass clusters similarly among bony fishes, amphibians, and reptiles, with birds and mammals together at a higher level, and cartilaginous fishes in between those two groups. Neuron numbers exist in the tens of millions for the ectothermic Classes, hundreds of millions in most mammals, and billions in most primates (Herculano-Houzel, 2017). To the extent that neural complexity can be related to degrees of consciousness, the range for both is great across the vertebrates overall.

The waking state, and therefore the capacity for awareness of the environment, has long been recognized as dependent on activation of a diffuse network of neurons emanating from the ancestral brain stem of vertebrates (Moruzzi and Magoun, 1949). This circuitry appears to be necessary for sensory awareness, but does not in itself provide the content of experience; that is assumed to be obtained from perceptions formulated in various regions of the cerebral cortex, which also holds distributed memory (Koch, 2012) and is reciprocally connected to various nuclei in the thalamus.

The neural substrate for complex cognitive functions that are associated with higher-level consciousness in mammals and birds are based on patterns of circuitry that are substantially less elaborated, with some components actually lacking, in reptiles, while the major thalamopallial circuits involving sensory relay nuclei are conspicuously absent in amphibians (Butler and Cotterill, 2006). Based on these criteria, the potential for higher-level consciousness in reptiles and amphibians appears to be lower than in birds and mammals.

A plausible blend of the above observations is that all vertebrates have the capacity for primary (sensory and affective) consciousness, but that the qualitative resolution and detail of conscious processes have risen as neocortical cell numbers have increased across different taxa.

While neural complexity may be marginal in insects (~1 million neurons in bees), many display behavior that goes well beyond simple reflexes and conditioned responses (Menzel, 2012; Giurfa, 2013; Chittka and Wilson, 2019). The complexity of the cephalopod brain, with >100 million neurons, exceeds that of frogs (Godfrey-Smith, 2016). Many arthropods and some cephalopods have complex neural regions involved in learning (hence in-memory storage)—“mushroom bodies” in insects, and the vertical lobe in octopi and squid. The behavior of arthropods and cephalopods is highly variable, in keeping with the variety of environments the animals occupy. Ecological psychologists argue that behavior—hence the nature of consciousness that is presumed to be needed to make the behavior possible—varies with the environmental constraints on each animal’s survival. Therefore, sensory consciousness may be created by diverse neural architectures, often through convergent evolution (Kaas, 2002; Emery and Clayton, 2004; Emery, 2006; Lisney and Collin, 2006; Lefebvre and Sol, 2008; Feinberg and Mallatt, 2016). It also follows, however, that the nature of that consciousness may vary greatly from that experienced by humans (Nagel, 1974).

Ancestral vertebrates and arthropods were present in the early Cambrian, 510 million years ago (mya). The earliest cephalopods date from about 490 mya. If primary consciousness was present in some members of all three groups, which are very distantly related, the origin of consciousness is ancient and, in all probability, evolved independently (Feinberg and Mallatt, 2016).

## Discussion

Modern science avoids subjectivity as much as possible, so the phenomenological, or subjective, nature of personal experience has always been the primary challenge to scientific research on consciousness. To that has been added centuries of introspection that treated consciousness as an entity, but one lacking material substance. Yet other subjective aspects of mental activity, like perception, emotion, learning, and dreaming have advanced progressively toward neuroscientific illumination. If consciousness were viewed more like those phenomena, as a *process*, perhaps the challenge to its scientific study would not seem so formidable.

The adaptive nature of consciousness, which focuses on the intimate interaction between body, brain, and environment, provides an ecological and evolutionary platform for the study of consciousness familiar to all biologists. The brain, and therefore the potential for consciousness, develops and functions in every species within a form of existence that is defined by the world it inhabits (Gallagher, 1997).

This article has argued that consciousness is more widespread than is generally believed, is generated by a diversity of neural substrates, has evolved independently several times, and appears over a range of degrees in a variety of forms (Feinberg and Mallatt, 2016). Technological advances in monitoring and visualizing brain activity in humans will surely illuminate human consciousness in ever-growing detail (Varela, 1996; Crick and Koch, 1998; Seth et al., 2006; Massimini et al., 2009; van Vugt et al., 2018). By superimposing the known electrophysiological correlates of place perception and motor activity (O’Keefe, 1990; Finkelstein et al., 2016; Moser et al., 2017) with established neuro indicators of awareness (Moruzzi and Magoun, 1949; Frith, 2002; Hameroff, 2010), a deeper understanding of the interaction between motor control and consciousness will be achieved (Stocker, 2016).

More attention should now be turned as well to the study of other animals in their natural environments, combining ethological techniques developed over the decades with increasingly sophisticated neuroscience methodologies (Morris, 2005; Gallagher and Zahavi, 2008; Boly et al., 2013; Reiter et al., 2017; Pennartz et al., 2019), to reveal indicators of consciousness in other species. Several other strategic and experimental approaches for assessing comparative cognition (including consciousness) are proposed in Irwin and Irwin (2020).

## Author Contributions

The author confirms being the sole contributor of this work and has approved it for publication.

## Conflict of Interest

The author declares that the research was conducted in the absence of any commercial or financial relationships that could be construed as a potential conflict of interest.
